# Oral adverse effects of head and neck radiotherapy with/without chemotherapy in a southern Brazil hospital

**DOI:** 10.1590/1807-3107bor-2025.vol39.008

**Published:** 2025-03-31

**Authors:** Joana Leticia Schorr, Felippe José Almeida Loureiro, Lauren Frenzel Schuch, Vivian Petersen Wagner, Vinicius Coelho Carrard, Matheus Neves, Marco Antonio Trevizani Martins, Manoela Domingues Martins

**Affiliations:** (a) Universidade Federal do Rio Grande do Sul – UFRGS, School of Dentistry, Department of Oral Pathology, Porto Alegre, RS, Brazil.; (b) Universidade Estadual de Campinas – Unicamp, Piracicaba Dental School, Department of Oral Diagnosis, Piracicaba, SP, Brazil.; (c) Universidad de la República, School of Dentistry, Montevideo, Uruguay.; (d) Universidade de São Paulo – USP, School of Dentistry, Departament of Stomatology, São Paulo, SP, Brazil.; (e) Universidade Federal do Rio Grande do Sul – UFRGS, School of Dentistry, Department of Preventive Dentistry, Porto Alegre, RS, Brazil.

**Keywords:** Radiotherapy, Squamous Cell Carcinoma of Head and Neck, Osteoradionecrosis, Oral Medicine

## Abstract

The aim of this study was to evaluate the main oral adverse effects and their relationship with dental care before radiotherapy (RT) and combined RT and chemotherapy (RT+CT). Additionally, we assessed the association of other risk factors with the development of these adverse effects. This paper is a retrospective cross-sectional analytical study of data from medical records of patients with head and neck cancer (HNC) who underwent RT or RT+CT attended at the stomatology unit in a southern Brazil hospital. The records of 78 patients with HNC were accessed. Demographic data, tumor characteristics, cancer treatment, follow-up data, adverse effects, and dental treatment prior to RT were evaluated individually for descriptive analysis. Kaplan-Meier survival curves were plotted. Risk factors were assessed using chi-square or Mann-Whitney test. The results showed that most patients were male (88.3%) in their 6^th^ decade of life. Tumors were mostly located in the oral cavity (52.6%) and squamous cell carcinoma was the main diagnosis (94.8%). Most cases were diagnosed in advanced stages (67.9%). RT was of 2D or 3D types in 93.6% of patients. Oral adverse effects were observed in 94.7% of patients treated with RT or RT+CT. Osteoradionecrosis (ORN), oral mucositis (OM), candidiasis, and xerostomia were the main complications. Patients who received 2D RT had higher risk of developing ORN and xerostomia compared to those who received 3D RT. The risk of developing ORN was higher in smokers compared to non-smokers and in patients who had not previously consulted a dentist. This study showed that patients who underwent RT for HNC have a high occurrence of oral complications. Dentists play a key role in the management of these oral side effects.

## Introduction

HNC, the seventh most prevalent cancer globally, encompasses various malignancies that affect regions like the oral and nasal cavities, pharynx, larynx, and salivary glands.^1,2^ Squamous cell carcinomas (SCC) account for about 90% of HNC cases, predominantly affecting men aged over 50.^2^ Major causal factors include tobacco and alcohol use, HPV for oropharyngeal carcinomas, and UV radiation for lip cancer.^3,4^ Early-stage HNC is typically treated with surgery or RT,^2,4^ but over 60% of cases are advanced, requiring multimodal approaches for improved cure rates while preserving functionality and quality of life.^5,6^ Surgical resection, often with neck dissection, followed by adjuvant RT or chemoradiotherapy (RT+CT), is preferred for oral cancer. In situations where surgery poses challenges or compromises long-term function, RT+CT stands as the primary curative treatment.^4-7^

Patients with HNC are typically given high doses of radiation (50 to 70 Grays) and several oral adverse effects (OAE) such as OM, xerostomia, radiation caries, ORN, trismus, and dysgeusia are common.^8^ Some of these adverse effects are considered acute and others of late onset, some are temporary while some are lifelong in cancer survivors.^8,9^ In addition, they have been linked to increased morbidity and mortality.^9^

The involvement of the dentist in the multidisciplinary team working with patients who will receive RT or RT+CT for HNC is key to prevent, minimize, and treat these side effects.^9,10^ Knowing the prevalence and risk factors of oral side effects can guide professionals when establishing protocols for standard care.^10^ Also, the importance of prior dental consultation justifies the inclusion of a dentist in the multidisciplinary team.^9,10^ Therefore, the aim of this study was to evaluate the main oral adverse effects and their relationship with dental care before RT or RT+CT through a retrospective analysis of medical records of HNC patients in a hospital in the south of Brazil. Furthermore, we assessed how other risk factors, such as the type of RT and patient habits, contribute to the development of these adverse effects.

## Methods

### Study design and ethics approval

A cross-sectional study was conducted to analyze medical records (2010-2019) from the Otorhinolaryngology and Stomatology Department at the Clinics Hospital of Porto Alegre, Brazil. The protocol was approved by the hospital's Ethics Committee (approval number: 39315220.3.0000.5327) and it adhered to STROBE guidelines for reporting observational studies.^11^

### Sample

This study was based on a convenience sample. A list of 614 patients who had outpatient consultations at the Otorhinolaryngology Service from 2010 to 2019 was previously filtered by the Medical Archive and Health Information Service (SAMIS) team.

### Inclusion criteria

Inclusion criteria were patients who received RT or RT+CT in the head and neck region attended by the stomatology unity of HCPA from January 2010 to December 2019. The patients were selected by the International Classification of Disease (ICD): Malignant neoplasm of mouth, unspecified (10 C06.9), Malignant neoplasm of larynx, unspecified (10 C32.9), Malignant neoplasm of the floor of the mouth (10 C04), and Malignant neoplasm of lip, oral cavity, and pharynx with invasive lesion (14.8).

### Exclusion criteria

**Figure 1:**
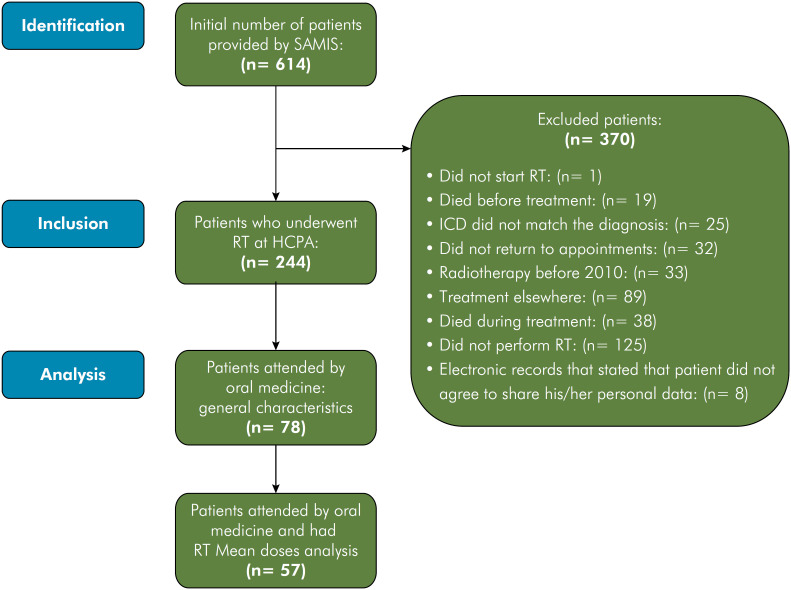
Flowchart showing the data collection process.

The study applied several exclusion criteria: 1. Patients who did not initiate RT; 2. Patients who died before treatment; 3. Patients with non-matching ICD diagnoses; 4. Patients failing to attend appointments; 5. Patients with prior RT history or treatment elsewhere; 6. Patients who died during HNC treatment; 7. Patients not receiving RT; and 8. Patients who refused data sharing. The flowchart in Figure 1 depicts the selection process. Among 614 patients, 371 were excluded, leaving 244 HNC patients who underwent RT or RT+CT. Seventy-eight were assessed by the oral medicine/stomatology team and 57 had full RT protocol data as demonstrated in the study flowchart.

### Data Collection

Data were managed in Microsoft Excel 2010^®^ by one author. Collected medical records included patient demographics (sex and age), habits (tobacco and alcohol consumption), diagnosis, tumor details (site and clinical stage), treatment specifics (surgery, RT, CT), doses to the mandible, maxilla, and parotid glands, follow-up, recurrence, metastasis, pre-RT consultation, oral adverse effects (OAE), and status (alive or dead). Records lacking adverse effects evaluation were deemed unreported. Only records mentioning OAE were analyzed.

### Data analysis

Descriptive and analytic statistics were conducted in SPSS version 25.0 (SPSS Inc, Armonk, USA). Continuous variables were expressed as mean, standard deviation, and range, while categorical variables were reported as absolute number and frequency. OAE occurrence was determined based on valid cases, excluding unclear records. Kaplan-Meier cumulative survival curves were plotted. The risk or protective factors were assessed via the association of categorical or continuous variables with adverse effects using Chi-square or Mann-Whitney test. Significant associations underwent logistic regression for odds ratio (OR) and 95% confidence interval (CI) calculation. All tests used a significance level of 5%.

## Results

### Demographic and clinical aspects

As shown in Table 1, seventy-eight patients were included, mostly male (n = 68/88.3%) in the sixth decade of life (mean 57.5). Oral cavity was the most frequent site (n = 41/52.6%) and SCC was the main diagnosis (n = 74 patients, 94.8%). Most cases were diagnosed in advanced stages (n = 53/67.9%).

**Table 1:** Number of patients (n) and relative frequencies (%) of clinical and pathological features.

Variable		n (%)
Sex		78 (100)
	Male	68 (88.3)
	Female	10 (11.7)
Age (years)		
	Mean ± SD	57.45 ± 11.12
	Min – max	mar/77
Tobacco consumption		78 (100)
	Smoker	40 (51.3)
	Former smoker	28 (35.9)
	Never smoked	10 (12.8)
Smoking load (pack/years) (n = 65)*		
	Mean ± SD	47.83 ±42.17
	Min–max	0–200
Alcohol consumption		78 (100)
	Former alcoholic	32 (41.0)
	Never/eventually	26 (33.3)
	Alcoholic	20 (25.7)
Histopathological diagnosis		78 (100)
	Squamous cell carcinoma	74 (94.8)
	Others**	4 (5.2)
Site of primary tumor		78 (100)
	Oral cavity	41 (52.6)
	Larynx	20 (25.6)
	Oropharynx	10 (12.8)
	Other than oral cavity, larynx and oropharynx	7 (9.0)
Clinical Stage		78 (100)
	I	3 (3.9)
	II	1 (1.3)
	III	12 (15.4)
	IV	53 (67.9)
	Missing	9 (11.5)

SD: standard deviation.

* Data were available for 65 of the 78 patients;

** Others: Verrucous carcinoma, rhabdomyosarcoma, adenoid cystic carcinoma, and sarcoma.

### Cancer treatment and follow-up

**Figure 2:**
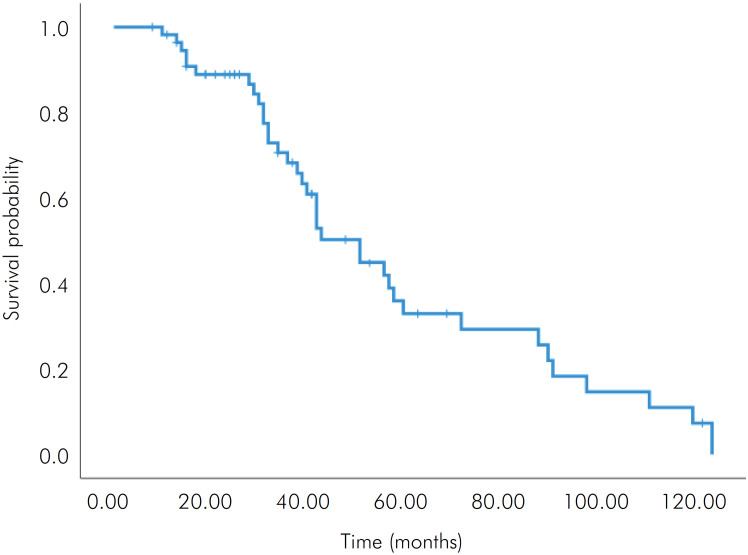
Kaplan-Meier survival curve of individuals with head and neck cancer treated with radiotherapy (RT) and combined RT and chemotherapy (RT+CT).

Table 2 presents data on the treatments performed and patients’ follow-up. Most patients (n = 41/52.6%) underwent RT+CT, while the others (n = 37/47.4%) were treated only with RT or surgery before or after RT. 3D RT was used in 52 patients (66.7%), conventional 2D RT in 21 (26.9%), and intensity modulated radiotherapy (IMRT) in 5 (6.4%) of the cases. Of the 78 patient records evaluated, 57 had information that allowed the calculation of the mean ± SD (min-max) RT dose. Parotid and mandible received the highest mean RT compared to the maxilla. After 2 years, thirty-one (54.4%) patients were alive, sixteen (28%) died, and ten (17.6%) were lost to follow-up. After 5 years, 10 (17.6%) were alive, 25 (43.9%) died, and 22 (38.5%) were lost to follow-up. Nineteen patients (24.3%) had recurrence during the follow-up and fourteen (17.9%) developed metastasis. Survival curves are shown in Figure 2.

**Table 2:** Number of patients (n) and relative frequencies (%) of treatment, radiotherapy dose, and follow up data.

Variable			n (%)
Treatment			78 (100)
	Radiotherapy associated with chemotherapy		41 (52.6)
	Surgery and radiotherapy/radiotherapy alone		37 (47.4)
Type of radiotherapy			78 (100%)
	3D		52 (66.7)
	Conventional 2D		21 (26.9)
	IMRT (with or without 3D)		5 (6.4)
Mean dDose for mMandible in cGy (n=57)*			
	Mean ± SD		4166.40 ± 1694.64
	Min – max		645.80 – 6478.10
Mean dDose for mMaxilla in cGy (n=57)*			
	Mean ± SD		2080.61 ± 1853.27
	Min – max		69.70 – 6665.70
Mean dDose for pParotid in cGy (n=57)*			
	Mean ± SD		4488.66 ± 1336.00
	Min – max		638.00 – 6341.30
Status (n=57)*			
	After 2 years		57 (100%)
		Alive	31 (54.4)
		Dead	16 (28.0)
		Loss to follow-up/Hospital discharge	10 (17.6)
	After 5 years		57 (100)
		Alive	10 (17.6)
		Dead	25 (43.9)
		Loss to follow-up/Hospital discharge	22 (38.5)
Recurrence			78 (100)
	No		58 (74.4)
	Yes		19 (24.3)
	Missing		1 (1.3)
Metastasis			78 (100%)
	No		64 (82.1)
	Yes		14 (17.9)

SD: standard deviation; cGy – centigray.

* Data were available for 57 of the 78 patients.

### Oral adverse effects (OAE)

**Figure 3:**
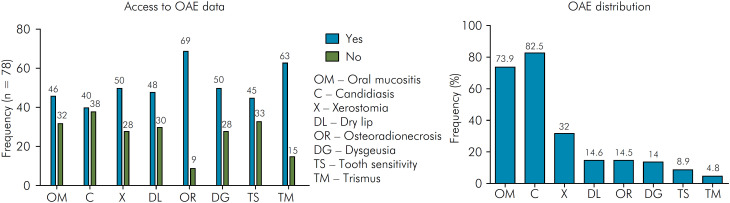
Frequency oral adverse effects. Of the 78 records, 46 contained information on oral mucositis, 40 on candidiasis, 50 on xerostomia, 48 on dry lip, 69 on osteoradionecrosis, 50 on dysgeusia, 45 on tooth sensitivity, and 63 on trismus, as shown on the left-hand side. From these data, the frequency (%) of patients who had OAE are shown on the right-hand side.

The oral side effects are described in Table 3 and presented in Figure 3. Of the 57 individuals included, 54 (94.7%) had some adverse effects from RT in the oral cavity. Most patients presented at least one adverse effect (n = 22/38.6%). Of the 46 medical records that reported the evaluation of OM, 34 (73.9%) mentioned clinical signs of OM. Forty medical records evaluated candidiasis status and 33 (82.5%) individuals had this condition. Fifty records evaluated xerostomia and 16 (32%) patients had this side effect.

**Table 3:** Number of patients (n) and relative frequencies (%) of dentist evaluation before RT or RT+CT and oral adverse effects (OAE) related to head and neck cancer treatment.

Variable		n (%)
Dentist consultation before RT or RT+CT treatment		78 (100%)
	Yes	47 (60.3)
	No	31 (39.7)
Presence of oral adverse effect (n = 57)*		57 (100%)
	Yes	54 (94.7)
	No	3 (5.3)
Number of oral adverse effects (n = 57)*		57 (100%)
	0	3 (5.3)
	1	22 (38.6)
	2	14 (24.6)
	3	16 (28.1)
	4	2 (3.5)
Oral adverse effect **		
	Oral mucositis (n = 46/78)	34/46 (73.9)
	Candidiasis (n = 40/78)	33/40 (82.5)
	Xerostomia (n = 50/78)	16/50 (32.0)
	Dry lip (n = 48/78)	7/48 (14.6)
	Osteoradionecrosis (n = 69/78)	10/69 (14.5)
	Dysgeusia (n = 50/78)	7/50 (14)
	Tooth sensitivity (n = 45/78)	4/45 (8.9)
	Trismus (n = 63/78)	3/63 (4.8)

* Data were available for 57 of the 78 patients.

** Data were analyzed by the assessment of the OAE among the 78 records. Frequency distributions are shown in Figure 3.

Table 4 illustrates the association between dental appointments before RT treatment and occurrence of OAE. Patients without a dental consultation had a 4.22 (0.98–18.06, p = 0.05)-fold increased chance of presenting ORN compared to patients who received dental care. In addition, patients who received 2D RT had a 2.59 (1.24– 5.41, p = 0.01)-fold increased risk of presenting ORN and a 2.69 (1.21–5.96, p = 0.01)-fold increased risk of presenting dry mouth/xerostomia compared with 3D (Table 5).

**Table 4:** Association between dental visit before treatment and prevalence of OAE.

Variable	Dentist consultation		p-value
	No (%)	Yes (%)	
Prevalence of any oral adverse effect (n = 57)	92.6	96.7	0.46
Prevalence of oral mucositis (n = 46)	63.2	81.5	0.19
Prevalence of candidiasis (n = 40)	83.3	81.8	0.61
Prevalence of tooth sensitivity (n = 45)	4.8	12.5	0.35
Prevalence of xerostomia (n = 50)	40.0	24.0	0.18
Prevalence of dry lip (n = 48)	16.7	12.5	0.50
Prevalence of trismus (n = 63)	0	7.7	0.23
Prevalence of osteoradionecrosis (n = 69)	25.0	7.3	0.04*
Prevalence of dysgeusia (n = 50)	12.5	15.4	0.54

* Statistically significant difference (Chi-square test).

**Table 5:** Association between radiotherapy type (2D or 3D) and prevalence of OAE.

Variable	Type of radiotherapy		p-value
	3D (%)	2D (%)	
Prevalence of any oral adverse effect (n = 52)	92.1	100	0.38
Prevalence of oral mucositis (n = 41)	73.5	57.1	0.32
Prevalence of candidiasis (n = 35)	82.1	71.4	0.43
Prevalence of tooth sensitivity (n = 40)	8.8	0	0.60
Prevalence of xerostomia (n = 46)	21.6	66.7	0.01*
Prevalence of dry lip (n = 43)	10.8	33.3	0.19
Prevalence of trismus (n = 58)	1.9	0	0.89
Prevalence of osteoradionecrosis (n = 64)	9.6	41.7	0.01*
Prevalence of dysgeusia (n = 45)	7.9	28.6	0.16

* Statistically significant difference (Chi-square test).

Patients who were current smokers had a 10.66 (1.26 – 89.62, p=0.02)-fold increased risk of presenting ORN compared to patients who had quit smoking or never smoked (Table 6).

**Table 6:** Association between osteoradionecrosis and site of primary tumor and smoking status.

Variable		Osteoradionecrosis		p-value
		No	Yes	
		n ((%	n ()	
Site				
	Oral cavity	26 (44.1)	10 (100)	< 0.001*
	Others	33 (55.9)	0 (0)	
Smoking status				
	Current smoker	27 (45.8)	9 (90)	0.01*
	Never / former smoker	32 (54.2)	1 (10)	

* Statistically significant difference (Chi-square test).

The radiation dose used in the patients analyzed did not affect the occurrence of adverse effects (Table 7). The presence of adverse effects in the oral cavity did not appear to change when adjuvant CT is combined with RT (p=0.31). Additionally, no correlation was found between adverse effects and nasoesophageal probe (Tables 8 and 9).

**Table 7:** Comparison of mean radiotherapy dose and the presence of oral adverse effects.

Variable	Mean dose of irradiation in cGy (maximum value)		p-value
	Not present	Present	
Any oral adverse effect (n = 43)	3669.0	5010.1	0.11
Oral mucositis (n = 39)	4441.8	5356.8	0.33
Candidiasis (n = 33)	5556.9	5285.9	0.17
Tooth sensitivity (n = 39)	5154.6	4395.3	0.28
Xerostomia (n = 41)*	4334.8	4203.7	0.60
Dry lip (n = 42)	4926.8	5006.8	0.88
Trismus (n = 57)	5011.9	5838.6	0.27
Osteoradionecrosis (n = 57)**	4063.7	5233.6	0.27
Dysgeusia (n = 43)	4887.8	5482.2	0.54

cGy: centigray.

* Parotid mean dose of irradiation was used for analysis.

** Mandible mean dose of irradiation was used for analysis.

Statistical test: Mann-Whitney.

**Table 8:** Association between chemotherapy during treatment and prevalence of oral adverse effects.

Variables	Adjuvant chemotherapy		p-value
	No	Yes	
	%	%	
Prevalence of any oral adverse effect (n = 57)	91.7	97.0	0.38
Prevalence of oral mucositis (n = 46)	88.2	65.5	0.08
Prevalence of candidiasis (n = 40)	73.3	88.0	0.22
Prevalence of tooth sensitivity (n = 45)	17.6	3.6	0.14
Prevalence of xerostomia (n = 50)	31.8	32.1	0.61
Prevalence of dry lip (n = 48)	10.0	17.9	0.37
Prevalence of trismus (n = 63)	3.7	5.6	0.60
Prevalence of osteoradionecrosis (n = 64)	13.3	15.4	0.54
Prevalence of dysgeusia (n = 50)	18.2	10.7	0.36

Statistical test: Chi-square.

**Table 9:** Association between use of nasoesophageal probe and prevalence of oral adverse effects.

Variable	Nasoesophageal probe		p value
	No	Yes	
	%	%	
Prevalence of any oral adverse effect (n = 57)	94.7	94.7	1.00
Prevalence of oral mucositis (n = 46)	71.4	75.0	0.53
Prevalence of candidiasis (n = 40)	80.0	83.3	0.57
Prevalence of tooth sensitivity (n = 45)	14.3	6.5	0.36
Prevalence of xerostomia (n = 50)	41.2	27.3	0.24
Prevalence of dry lip (n = 48)	6.7	18.2	0.28
Prevalence of trismus (n = 63)	5.9	4.3	0.61
Prevalence of osteoradionecrosis (n = 64)	16.7	14.0	0.52
Prevalence of dysgeusia (n = 50)	17.6	12.1	0.44

Statistical test: Chi-square.

## Discussion

The aim of RT, a pivotal treatment for HNC, is to eradicate malignant cells while preserving organs and providing adjuvant or definitive care.^7^ However, it often induces adverse effects, influenced by various factors.^7,12^ In our study, we analyzed the records of 78 HNC patients undergoing RT or RT+CT, focusing on OAE and their correlation with prior dental care. Our findings revealed a significant occurrence of OAE and highlighted the association between inadequate dental care, RT modality, and OAE occurrence, particularly in ORN development.

Our sample comprised predominantly male patients (88.3%) in their sixth decade, diagnosed with advanced-stage SCC (94.8%), most commonly affecting the oral cavity (52.6%), followed by the larynx (25.6%), and oropharynx (10.8%). These findings align with the literature, indicating that over 60% of HNC cases are advanced neoplasms, requiring multimodal approaches including RT.^6,13^

The challenge of RT is to maximize radiation doses to the tumor while minimizing normal tissue exposure, increasing cure rates and reducing morbidity.^14,15^ Techniques such as traditional RT and IMRT distribute radiation differently.^14^ Conventional RT employs orthogonal skeletal views and multiple beams, potentially irradiating large tissue volumes.^14^ In contrast, MRI-guided RT allows precise tumor and normal tissue delineation, optimizing dose distribution.^14,15^ In our sample, 94.7% of patients developed OAE and the majority (93.6%) received conventional 2D or 3D RT. Only 6.4% of the patients received IMRT. This could be explained by the fact that at the HCPA hospital, IMRT was initiated in 2019, which was the last year of the patients included in the present study. Parotid glands and mandibles received a higher mean RT value compared to maxillas. No difference in OM percentage was observed between 2D RT and 3D. This result was similar to that of Ghosh-Laskar et al.^16^ that found no significant differences in incidence of any of the other acute toxicities, such as mucositis, in a randomized controlled trial to compare 3D RT to IMRT. However, our study showed a significant association between RT type (2D) and occurrence of xerostomia and ORN. These results are in accordance with the literature that clearly demonstrates that patients with HNC are submitted to high RT doses, which are associated to several side effects that can be chronic, debilitating, and challenging complications, such as xerostomia and ORN.^9,17,18^ To mitigate them, IMRT and proton therapy can be used, which offer a better dose distribution and spare healthy tissues to a greater extent.^8,19^ Gupta et al.^12^ found less acute grade 2 or worse xerostomia with IMRT compared to 3D RTR (59% vs 89%; p = 0.009). Like xerostomia, the use of 3D RT or IMRT protects bones from excessive secondary radiation.^16,20^

RT has a significant impact on oral tissues, including oral mucosa, salivary glands, masticatory muscles, and jaw bones.^17^. In the present study, the most relevant adverse effects in terms of their occurrence and impact on patients were OM, candidiasis, xerostomia, and ORN. Of the 78 patients included, 54 (60.3%) had a previous appointment with a dentist for oral cavity assessment and received oral health care procedures or orientations. Our results also demonstrated that dental care before RT or RT+CT had an important impact on the development of ORN. Patients without a dental consultation had a 4.22-fold increased chance of developing ORN compared to patients who received dental care. For other side effects such as OM and candidiasis, dental care had no impact on their frequency, probably altering the severity of the manifestation, which was not evaluated in the study. In a cohort of patients with similar conditioning regimens before hematopoietic stem cell transplantation, Coracin et al.^21^ observed that oral health parameters, including dental plaque and periodontal state, were predictive of the occurrence and severity of OM.^21,22^

Radiation-induced OM was a common acute complication, affecting 79% of our study patients. This was similar to previous reports ranging from 89 to 100% among RT or RT+CT recipients. Unfortunately, our records lacked OM grading data, which are crucial for assessing the impact of dental care on severity.^22-24^ OM development in RT is multifactorial, influenced by treatment parameters and individual susceptibility.^24,25^ Since 2010, our oral medicine team has provided preventive and therapeutic measures like photobiomodulation for HNC patients undergoing RT.^25^ However, comprehensive evaluation remains challenging due to missing information.

RT in the head and neck region can damage salivary glands, leading to a permanent decreased saliva production, characterizing the xerostomia.^9,10^ In our study, xerostomia was as a significant side effect, particularly associated with 2D RT. Conversely, patients undergoing 3D RT exhibited lower incidence of xerostomia. This condition can affect speech and swallowing and increase the risk of dental problems.^9,10^ Advanced techniques like IMRT have decreased xerostomia risk in HNC patients.^26-28^ Choi et al.^10^ demonstrated the efficacy of IMRT in reducing parotid gland radiation doses without compromising therapeutic outcomes. Their study suggests a dose limit of 26–30 Gy for preserving parotid gland function in HNC patients treated with IMRT.

The most important results of the present study was the association of ORN with 2D RT. RT-induced changes in bone, including reduced blood flow and fibrosis, are known to increase susceptibility to ORN, which is characterized by ischemic necrosis of irradiated bone tissue.^9,10,20^ Like xerostomia, 3D RT or IMRT treatment also protects bone from excessive secondary radiation.^20^ Patients without prior RT dental consultation had a 4.22-fold increased chance of presenting ORN compared to patients who received dental care. Also, patients who were current smokers had a 10.66-fold increased risk of presenting ORN compared to patients who quit smoking or never smoked. In general, dental hygiene practices have a crucial role in mitigating the risk of ORN by promoting optimal oral health, minimizing the potential for infection, and enhancing tissue healing.^10,20^ Smoking is known to have detrimental effects on overall health and can adversely affect wound healing and tissue vascularization.^20^ In the context of RT, smoking can exacerbate the negative effects of radiation on oral tissues, making them more susceptible to ORN.^10,20^ Furthermore, studies have shown that smoking cessation prior to or during radiation therapy can significantly reduce the risk of ORN.^20,30^

Collaboration among radiation oncologists, head and neck surgeons, and dentists is vital for preventive measures and dental care. Supportive measures like oral hygiene, rinses, pain management, and nutrition contribute to patient comfort.^10,30^ Choi *et al*.^10^ evaluated the economic impact of the participation of dentists in the oncological team for the prevention and treatment of oral complications from HNC therapy. Their results demonstrated that the involvement of dentists resulted in lower costs and shorter treatment duration for acute complications. In the group of patients who visited dentists, the average treatment cost for total acute complications was $974 in US dollars (95%CI: 0.904–1,045) with a treatment duration of 14.57 (95%CI: 14.14– 5.00) days. In contrast, the average treatment cost for the patients who did not visit dentists was $2,089 (USD) (95%CI: 1,991–2,186) with a treatment duration of 25.83 (95%CI: 25.52–26) days. Dental hygiene practices are often integrated into the overall management strategy to minimize the risk of ORN and optimize treatment outcomes.

Performing dental treatment before starting RT or CT in the head and neck region is extremely important to guarantee the patient's oral health and minimize complications during cancer treatment.^10,31^ Proper oral health reduces the risk of infections and OAE, which can be worsened by anticancer therapy10. However, radiation therapy cannot be unnecessarily delayed as it diminishes a patient's prognosis^31^. The multidisciplinary team should plan what is best for each patient in the different cases.

Retrospective studies offer valuable insights into healthcare and medical research, yet they come with inherent limitations. In our study, there were issues with the availability and quality of data from the medical records, which could impact conclusions and introducing bias. Inaccurate timing recording may hinder establishing cause-effect relationships. Additionally, the generalizability of findings may be limited due to sample characteristics such as demographics, treatment protocols, and healthcare settings. Despite these constraints, retrospective analyses are still valuable for understanding healthcare dynamics, emphasizing the need for cautious interpretation and consideration of study limitations.

## Conclusion

The study underscores the importance of interdisciplinary care of HNC patients, especially regarding the adverse effects of RT alone or combined with CT on the oral cavity. Consulting with a specialist dentist before and during treatment, particularly concerning ORN development, is emphasized. Thus, integrating oral medicine into oncology teams is crucial for enhancing patient care in HNC treatment.

## References

[1] Alsahafi E, Begg K, Amelio I, Raulf N, Lucarelli P, Sauter T (2019). Clinical update on head and neck cancer: molecular biology and ongoing challenges. Cell Death Dis.

[2] WHO Classification of Tumours Editorial Board (2022). Head and neck tumours.

[3] Machiels JP, René Leemans C, Golusinski W, Grau C, Licitra L, Gregoire V (2021). Reprint of "Squamous cell carcinoma of the oral cavity, larynx, oropharynx and hypopharynx: EHNS-ESMO-ESTRO Clinical Practice Guidelines for diagnosis, treatment and follow-up". Oral Oncol.

[4] Pfister DG, Spencer S, Adelstein D, Adkins D, Anzai Y, Brizel DM (2020). Head and Neck Cancers, Version 2.2020, NCCN Clinical Practice Guidelines in Oncology. J Natl Compr Canc Netw.

[5] Pisani P, Airoldi M, Allais A, Aluffi Valletti P, Battista M, Benazzo M (2020). Metastatic disease in head & neck oncology. Acta Otorhinolaryngol Ital.

[6] Brana I, Siu LL (2012). Locally advanced head and neck squamous cell cancer: treatment choice based on risk factors and optimizing drug prescription. Ann Oncol.

[7] Bernier J, Cooper JS, Pajak TF, Glabbeke M, Bourhis J, Forastiere A (2005). Defining risk levels in locally advanced head and neck cancers: a comparative analysis of concurrent postoperative radiation plus chemotherapy trials of the EORTC (#22931) and RTOG (# 9501). Head Neck.

[8] Siddiqui F, Movsas B (2017). Management of radiation toxicity in head and neck cancers. Semin Radiat Oncol.

[9] Sroussi HY, Epstein JB, Bensadoun RJ, Saunders DP, Lalla RV, Migliorati CA (2017). Common oral complications of head and neck cancer radiation therapy: mucositis, infections, saliva change, fibrosis, sensory dysfunctions, dental caries, periodontal disease, and osteoradionecrosis. Cancer Med.

[10] Choi SE, Choudhary A, Sonis S, Villa A (2021). Benefits of the involvement of dentists in managing oral complications among patients with oral cavity and oropharyngeal cancer: an analysis of claims data. JCO Oncol Pract.

[11] Vandenbroucke JP, Elm E, Altman DG, Gøtzsche PC, Mulrow CD, Pocock SJ (2007). Strengthening the Reporting of Observational Studies in Epidemiology (STROBE): explanation and elaboration. PLoS Med.

[12] Gupta T, Kannan S, Ghosh-Laskar S, Agarwal JP (2018). Systematic review and meta-analyses of intensity-modulated radiation therapy versus conventional two-dimensional and/or or three-dimensional radiotherapy in curative-intent management of head and neck squamous cell carcinoma. PLoS One.

[13] Sung H, Ferlay J, Siegel RL, Laversanne M, Soerjomataram I, Jemal A (2021). Global Cancer Statistics 2020: GLOBOCAN estimates of incidence and mortality worldwide for 36 cancers in 185 countries. CA Cancer J Clin.

[14] Kirthi Koushik AS, Harish K, Avinash HU (2013). Principles of radiation oncology: a beams eye view for a surgeon. Indian J Surg Oncol.

[15] Alterio D, Marvaso G, Ferrari A, Volpe S, Orecchia R, Jereczek-Fossa BA (2019). Modern radiotherapy for head and neck cancer. Semin Oncol.

[16] Ghosh-Laskar S, Yathiraj PH, Dutta D, Rangarajan V, Purandare N, Gupta T (2016). Prospective randomized controlled trial to compare 3-dimensional conformal radiotherapy to intensity-modulated radiotherapy in head and neck squamous cell carcinoma: long-term results. Head Neck.

[17] Jham BC, Freire ARS (2006). Oral complications of radiotherapy in the head and neck. Braz J Otorhinolaryngol.

[18] Mallick I, Waldron JN (2009). Radiation therapy for head and neck cancers. Semin Oncol Nurs.

[19] Moreno AC, Frank SJ, Garden AS, Rosenthal DI, Fuller CD, Gunn GB (2019). Intensity modulated proton therapy (IMPT): the future of IMRT for head and neck cancer. Oral Oncol.

[20] Moreno AC, Frank SJ, Garden AS, Rosenthal DI, Fuller CD, Gunn GB (2019). Intensity modulated proton therapy (IMPT): the future of IMRT for head and neck cancer. Oral Oncol.

[21] Beaumont S, Bhatia N, McDowell L, Fua T, McCullough M, Celentano A (2021). Timing of dental extractions in patients undergoing radiotherapy and the incidence of osteoradionecrosis: a systematic review and meta-analysis. Br J Oral Maxillofac Surg.

[22] Coracin FL, Santos PS, Gallottini MH, Saboya R, Musqueira PT, Barban A (2013). Oral health as a predictive factor for oral mucositis. Clinics (Sao Paulo).

[23] Pai RR, Ongole R, Banerjee S, Prasad K, George LS, George A (2019). Oral Care Protocol for Chemotherapy- and Radiation Therapy-Induced Oral Complications in Cancer Patients: study Protocol. Asia Pac J Oncol Nurs.

[24] Liu S, Zhao Q, Zheng Z, Liu Z, Meng L, Dong L (2021). Status of treatment and prophylaxis for radiation-induced oral mucositis in patients with head and neck cancer. Front Oncol.

[25] Trotti A, Bellm LA, Epstein JB, Frame D, Fuchs HJ, Gwede CK (2003). Mucositis incidence, severity and associated outcomes in patients with head and neck cancer receiving radiotherapy with or without chemotherapy: a systematic literature review. Radiother Oncol.

[26] Zadik Y, Arany PR, Fregnani ER, Bossi P, Antunes HS, Bensadoun RJ (2019). Systematic review of photobiomodulation for the management of oral mucositis in cancer patients and clinical practice guidelines. Support Care Cancer.

[27] D’Hondt E, Eisbruch A, Ship JA (1998). The influence of pre-radiation salivary flow rates and radiation dose on parotid salivary gland dysfunction in patients receiving radiotherapy for head and neck cancers. Spec Care Dentist.

[28] Ship JA, Eisbruch A, D’Hondt E, Jones RE (1997). Parotid sparing study in head and neck cancer patients receiving bilateral radiation therapy: one-year results. J Dent Res.

[29] Nutting CM, Morden JP, Harrington KJ, Urbano TG, Bhide SA, Clark C (2011). Parotid-sparing intensity modulated versus conventional radiotherapy in head and neck cancer (PARSPORT): a phase 3 multicentre randomised controlled trial. Lancet Oncol.

[30] Münter MW, Karger CP, Hoffner SG, Hof H, Thilmann C, Rudat V (2004). Evaluation of salivary gland function after treatment of head-and-neck tumors with intensity-modulated radiotherapy by quantitative pertechnetate scintigraphy. Int J Radiat Oncol Biol Phys.

[31] Bonan PR, Lopes MA, Pires FR, Almeida OP (2006). Dental management of low socioeconomic level patients before radiotherapy of the head and neck with special emphasis on the prevention of osteoradionecrosis. Braz Dent J.

[32] Arya L, Brizuela M (2023). Oral management of patients undergoing radiation therapy StatPearls.

